# Case report: Severe chronic spontaneous urticaria successfully treated with omalizumab and dupilumab 

**DOI:** 10.5414/ALX02382E

**Published:** 2023-01-03

**Authors:** Viktoria Puxkandl, Wolfram Hoetzenecker, Sabine Altrichter

**Affiliations:** 1 Department of Dermatology, Comprehensive Allergy Center, Kepler University Hospital Linz & Johannes Kepler University, Linz, Austria, and; 2 Departement of Dermatology and Allergology, Charité – Universitätsmedizin Berlin, Berlin, Germany

**Keywords:** chronic spontaneous urticaria, omalizumab, dupilumab, mast cells

## Abstract

Urticaria is a disease triggered by mast cells and characterized by recurrent symptoms such as wheals and/or angioedema. Most patients benefit from treatment with antihistamines (AH) or, if not effective enough, from additional therapy with omalizumab, which leads to significant symptom relief. Here we present a patient with therapy-resistant chronic spontaneous urticaria treated with AH, omalizumab, and glucocorticosteroids. The subsequent recommended therapy according to the current guideline, cyclosporine A, was contraindicated in this patient. Therefore, therapy with dupilumab was initiated, resulting in a complete control of symptoms. In this case report, we present a case of successful dual therapy with omalizumab and dupilumab.

## Case report 

Urticaria is a disease triggered by mast cells and characterized by recurrent symptoms such as itchy wheals and/or angioedema. When symptoms persist for more than 6 weeks, it is defined as chronic urticaria (CU). In the absence of an identifiable trigger, CU is classified as chronic spontaneous urticaria (CSU) [1]. 

CU can be very debilitating, and patients’ quality of life can be extremely low [2]. In the current guideline, the treatment goal is complete symptom control [1]. Second-generation antihistamines (AH) are recommended as first-line therapy, even at higher-than-approved doses. If symptoms can only be incompletely controlled, add-on therapy with omalizumab (monoclonal antibody against IgE) is recommended. Another effective therapy in patients with an antihistamine-resistant CU may be cyclosporine, which is currently recommended as third-line therapy [1]. Beyond the guideline, several drugs approved for other indications have been suggested as potentially effective [2]. Among them, dupilumab (monoclonal antibody against IL-4 and IL13 receptor α) was proposed as a possible treatment alternative [3, 4]. To date, several case reports have shown the efficacy of dupilumab therapy in CSU patients, mostly in patients with concurrent atopic dermatitis [5]. In addition, a case report of a patient with CSU and atopic dermatitis demonstrated successful simultaneous therapy with omalizumab and dupilumab for both conditions [6]. 

Treatment failure of AH and omalizumab in CSU has been described previously. More frequently, patients failed to respond to omalizumab therapy if they had a low total IgE and/or elevated IgG anti-TPO antibodies [7]. 

Here, we describe a patient with therapy-resistant CSU, without evidence of known markers for poor treatment response, who experienced symptom relief with a dual therapy of omalizumab and dupilumab. 

In August 2021, a 68-year-old woman presented with recurrent wheals and reported episodes of facial angioedema that had begun 6 months earlier. On initial clinical examination, she presented with wheals scattered over the entire body with no current evidence of angioedema. Her medical history consisted of NSAIDs intolerance (ibuprofen) and hypertension. Clinically, the diagnosis of CSU was made. Laboratory testing revealed a total IgE of 111 kU/L and no evidence of IgG anti-TPO autoantibodies (Table 1). 

The patient reported prior therapy with second-generation AH, which did not relieve symptoms. Because second-generation AH did not result in a response at doses of up to 4 times daily, systemic therapy with omalizumab was initiated (300 mg SC every 4 weeks). 

Only 3 days after the first injection, the patient presented with pronounced urticaria (> 50 wheals) and pruritus (UCT = 0) despite the maximum dose of AH (desloratadine 4 times daily). To provide the patient with relief, a glucocorticosteroid therapy (starting with prednisolone 50 mg/day) was initiated. Unfortunately, this therapy also failed to provide symptom relief after 2 days, so glucocorticosteroid dose was increased (prednisolone 75 mg/day), and the patient became symptom free. Over the next 20 days, the dose was reduced to 5 mg/day, after which symptoms recurred. At the next visit, the patient remained at prednisolone 10 mg/day for partial relief and was taking AH (desloratadine) 4 times daily, but still exhibited severe wheals (> 50 wheals). According to the guideline, omalizumab therapy was increased to 450 mg per month. 

Thereafter, she was only symptom-free for a few days when the hives recurred and the patient had new episodes with angioedema. However, following the therapeutic algorithm with the next recommended therapy with cyclosporine A was contraindicated due to a chronic renal insufficiency. 

At the next follow-up (November 2021), a skin biopsy was taken to rule out other differential diagnoses, but the histologic examination showed no evidence of urticaria vasculitis or a specific increased cytokine expression (Table 1). 

Six months after starting omalizumab treatment with additional glucocorticosteroids (5 mg/day) and AH therapy, the patient still had wheals (> 50 wheals) but no new angioedema episodes (UCT 7 points). Repeated attempts to discontinue glucocorticosteroids failed. 

Due to the limited therapeutic options of therapy-resistant CSU, the patient’s advanced age and concomitant diseases, additional therapy with dupilumab was initiated (600 mg initial injection – followed by 300 mg every 2 weeks). 

After the third injection of dupilumab, the patient was free of symptoms (no new wheals or angioedema; UCT = 16). Maintenance therapy with glucocorticosteroids was discontinued without recurrence of symptoms. In addition, AH therapy was reduced to twice daily. The patient did not experience any adverse effects during dual therapy with omalizumab and dupilumab. 

Based on the significant improvement and efficacy, omalizumab therapy was reduced back to the standard dose of 300 mg/4 weeks, and the patient remained on symptom control. 

Re-evaluation of the patient and therapy is planned after 6 months, and further stepping down of the therapy omalizumab therapy is planned if disease control persists. 

In summary, to our knowledge this is the first report of an adjunctive therapy of dupilumab in combination with omalizumab achieving complete control of clinical symptoms in a patient who had no history of manifest atopic dermatitis. The exact role of IL-4 and IL-13 in CSU is not clear, but these cytokines play a crucial role in IgE class switching, so blockade may be beneficial in CU patients [3, 8]. Interestingly, a recent report demonstrated long-term remission in several cases, suggesting a possible disease-modifying effect of dupilumab therapy [9]. 

A placebo-controlled trial of dupilumab for the treatment of CSU has been conducted, but final results have not yet been published [10]. However, dupilumab could be a future treatment option for CSU as an alternative or additional therapy. 

## Ethics approval and consent to participate 

The patient gave full informed consent on the use of her medical history and all utilized pictures for this case report. 

## Funding 

No funding was received for the study and the preparation of this manuscript. 

## Conflict of interest 

The authors have no conflict of interest to declare that are relevant to the content of this article. 

Outside this topic: Sabine Altrichter has conducted studies for / was advisor for / was speaker for AstraZeneca, Allakos, ALK, CSLBehring, LeoPharma, Moxie, Novartis, Sanofi, Takeda, and Thermofisher. Wolfram Hoetzenecker has conducted studies for / was advisor for / was speaker for Novartis, Eli Lilly, Bencard, ALK, Leo Pharma, Kyowa Kirin, Takeda, Sanofi-Aventis, and AbbVie. 

**Table 1.** Clinical keypoints.

Clinical exam	
Weight	93 kg
BMI	35.8 kg/m^2^
UCT at first presentation (August 2021)	0 points
UCT after treatment with Dupilumab and Omalizumab (August 2022)	16 points
Laboratory values (September 2021)	
Total IgE	111 IU/mL
Basophils	0.01 G/L
Eosinophils	0.02 G/L
IgG-anti-TPO	< 28 IU/mL
Histological findings (November 2021)	Very mild perivascular superficial lymphocytic inflammatory infiltrate, without neutrophils or nuclear dust. Scattered telangiectasia. MPO staining without pathological findings. Direct immunofluorescence without pathological findings. Diagnosis: Urticaria No evidence of urticarial vasculitis or specifically elevated cytokine expression.

BMI = body mass index; UCT = urticaria control test; TPO = thyreoperoxidase antibody; MPO = myeloperoxidase.
